# Association between the person-centered maternity care experience and mental health after delivery in urban and rural Dhading, Nepal: a cross-sectional study

**DOI:** 10.1186/s12884-023-05709-z

**Published:** 2023-05-30

**Authors:** Yuri Tomita, Junko Kiriya, Ram Chandra Silwal, Ken Ing Cherng Ong, Akira Shibanuma, Masamine Jimba

**Affiliations:** 1 Department of Community and Global Health, Graduate School of Medicine, The University of Tokyo, Tokyo, Japan; 2 Green Tara Nepal, Kathmandu, Nepal

**Keywords:** Person-centered maternity care, Depressive symptoms, Mental well-being, Nepal

## Abstract

**Background:**

Person-centered maternity care is a component of quality care, which includes effective communication, respect, and dignity. Supportive care has a positive effect on mothers’ perinatal experience. In contrast, negative childbirth experiences can cause psychological problems. However, the impact of person-centered maternity care experience on mothers’ mental health after delivery remains unknown. Therefore, in this study, we examined the association between person-centered maternity care experience at healthcare facilities and maternal mental health after delivery among Nepali women.

**Methods:**

We conducted a cross-sectional study in urban and rural areas in Dhading District, Nepal. Participants were women who gave birth at public healthcare facilities, and their baby’s age was between 1 and 12 months. After purposively selecting the target areas, we recruited the women from July to August 2019 and interviewed them using questionnaires. We conducted multiple regression analyses to analyze the association between delivery care experience and depressive symptoms and the association between delivery care experience and mental well-being.

**Results:**

In total, 595 women were included in the data analysis. The experience of better person-centered maternity care was associated with lower depressive symptom scores in urban (unstandardized coefficient [B]= − 0.09, *p* < 0.001) and rural areas (B= − 0.10, *p* < 0.001). Moreover, the experience of better person-centered maternity care was associated with higher mental well-being scores in both urban (B= 0.30, *p* < 0.001) and rural areas (B= 0.15, *p* = 0.017).

**Conclusions:**

Person-centered maternity care was associated with lower depressive symptom scores and higher mental well-being, regardless of the setting in Nepal. Person-centered maternity care during childbirth can potentially improve mental health after delivery. Maternity care should be improved with more attention to person-centered maternity care aspects.

**Supplementary Information:**

The online version contains supplementary material available at 10.1186/s12884-023-05709-z.

## Background

Improving the overall quality of maternal healthcare is the key to ensuring positive health outcomes [1, 2]. Most preventable deaths and disabilities during childbirth can be reduced by health services providing quality care [3]. However, the utilization of quality maternity services is restricted in some resource-limited settings, especially in sub-Saharan Africa and South Asia [4]. In these settings, women reported the barriers to using quality services at a facility, including socio-cultural norms, limited access, financial problems, and a negative perception of quality care [5]. Several women were reluctant to undergo a facility delivery due to mistreatment, verbal abuse, rudeness, and neglect [5].

To overcome these barriers, person-centered maternity care (PCMC) has been introduced in several countries. PCMC is the component of quality care, which is provided according to the preferences and aspirations of users [6]. Such care includes dignity, autonomy, privacy/confidentiality, communication, social support, supportive care, and trust [7]. PCMC allows women to make decisions for treatment, maintain their dignity, and improve their capabilities [6].

To implement PCMC, several supportive care or dignity interventions have been conducted. They include consultations with trained midwives or therapy groups; such care showed positive effect on women’s perinatal experience [8]. Those effects decreased women’s anxiety and birth-related concerns [8]. However, some women experience psychological birth trauma, and one of the risk factors is poor interaction with healthcare providers [9]. Approximately 20% of women experience mental difficulties after childbirth in low- and middle-income countries, which is higher than in high-income countries [10, 11]. Therefore, maternal mental health management is required after delivery, especially in resource-limited settings [12].

Nepal, one of the lower-middle-income countries in South Asia, is now focusing on high-quality health systems [13, 14]. Although maternity care usage has increased in the past 20 years, the facility delivery rate still has room for improvement [14]. One barrier to facility delivery was women’s perception of healthcare providers’ disrespectful care and the quality of health services [15, 16]. Women’s intentions for future maternity care utilization were influenced by the waiting time, received information, and overall care at the facility [17]. Despite this, women were dissatisfied with the physical resources and interpersonal aspects, such as compassion, respect, and honesty at public hospitals in Nepal [18]. Thus, a better quality of maternity care that considers women’s needs and satisfaction is required.

PCMC is also necessary to prevent mothers’ mental problems and improve their well-being. Nowadays, postpartum depressive symptoms are gradually gaining attention in Nepal [19, 20]. However, how the childbirth experiences at healthcare facilities, particularly with PCMC, are associated with women’s mental health after delivery in Nepal remains unknown. Moreover, only a few studies were conducted on the difference in maternal mental health between urban and rural settings in Nepal. Different risks were reported for postpartum depression, depending on the place and circumstances of residence [21].

Therefore, this study was conducted to examine the association between PCMC experiences at healthcare facilities and maternal mental health after delivery among women living in Dhading, Nepal. This study also explored the difference between urban and rural communities in the association of PCMC with maternal mental health after delivery.

## Methods

### Study design and settings

We conducted a cross-sectional study in Dhading District, Nepal. Dhading belongs to Bagmati Province and is adjacent to Kathmandu, the capital of Nepal. According to the preliminary Population Census 2021 result, the population of Dhading is 336,067 [22]. Its population density is 168 per square kilometer, ranked 38th among all 77 districts in Nepal [22]. As the study site, we covered all 2 municipalities and 5 out of 11 rural municipalities. These municipalities and rural municipalities had both urban and rural areas at the ward level based on the classification of the Central Bureau of Statistics in Nepal. Ward is the smallest local unit in Nepal. This classification was also used in the Demographic Health Survey in 2016 [14]. Rural areas consisted of smaller wards with an average of 104 households per ward. Urban areas consisted of larger wards with an average of 800 households per ward [14].

### Participants

We recruited mothers who were 15–49 years old, had a child aged 1–12 months, and gave birth at public healthcare facilities located in Dhading. The child’s age was set at 1–12 months because many women experience postpartum blues or mental disorders immediately after childbirth [23, 24]. We excluded women who were referred to a tertiary referral hospital in another district or could not speak the Nepali language. We also excluded women living in remote areas, where access was difficult.

We calculated the sample size using Open Epi version 3.01 for the cross-sectional study. We calculated the number based on the previous study with a similar study design conducted by Abbott et al. [25], with 80% power and the significance level set at 5%. The required sample size was 310 for each urban and rural setting; thus, the total expected sample size was 620.

We recruited women through the following procedure. First, we selected 5 out of 13 healthcare facilities in urban areas and then 6 out of 49 facilities in rural areas. These were purposively selected based on the number of deliveries in the previous fiscal year. Many healthcare facilities had fewer than 30 deliveries within the previous fiscal year, so we excluded these facilities. Hence, the included health facilities had more deliveries than others. Also, we considered the location of healthcare facilities and excluded the regions that were difficult to visit due to the feasibility of data collection; therefore, 10 healthcare facilities were excluded even though they had more than 30 deliveries within the previous fiscal year. Consequently, we included different types of public healthcare facilities: district hospitals, primary health centers, and health posts. The health post is the primary level public health center, which provides basic health services [26]. Thereafter, we estimated the number of eligible women for each ward where the healthcare facilities were located to determine the required sample size per healthcare facility. Healthcare facility staff assisted us in obtaining the list of eligible women and their living wards. Finally, we visited wards to find these eligible women by asking local people about the areas of residence for the eligible women on the list. We finished the recruitment at the ward after enlisting the required number of eligible women purposively.

### Variables and assessment

#### Exposure variable

The exposure variable was the mothers’ perception of PCMC experience during childbirth. We used the PCMC scale for the assessment [27]. This scale is validated and has been used in several low- and middle-income countries [28]. It measures mothers’ experience in a healthcare facility during their latest delivery, and especially how they were treated at the facility. The PCMC scale consists of 30 items with 3 sub-scales: dignity and respect, communication and autonomy, and supportive care. Each item was coded from 0 to 3, and the total scores ranged from 0 to 90 points. For the following questions, reverse coding was conducted so that high scores equaled high PCMC: 1, 21, 22, and 26. When the participant answered “not applicable (N/A)”, it was scored with the highest point as suggested in the guideline of the questionnaire. A higher score indicates women who experienced better PCMC. Also, the Cronbach’s alpha was calculated for each scale to assess internal consistency. The Cronbach’s alpha was 0.93 for the PCMC scale.

#### Outcome variables

The outcome variables were maternal depressive symptoms and mental well-being after delivery. We set two outcome variables to measure the negative and positive sides of maternal mental health. The Edinburgh Postnatal Depression Scale (EPDS) was used to measure depressive symptoms [29]. EPDS is a validated scale for screening postnatal depression, and we used the validated Nepali version of EPDS [29, 30]. EPDS has 10 items, each of which scored from 0 to 3; therefore, its total score ranges from 0 to 30. Reverse coding was conducted for the following questions: 3, 5–10. The cut-off point for the possibility of having depressive symptoms was EPDS score > 12, according to previous studies [29, 30]. In this study, the Cronbach’s alpha was 0.85 for EPDS.

The Warwick-Edinburgh Mental Wellbeing Scale (WEMWBS) was used to assess mental well-being [31]. WEMWBS is a validated scale that is used in a variety of settings, including low- and middle-income countries [31]. Although the target population was not mothers, WEMWBS was validated in Nepal previously [32]. WEMWBS consists of 14 positively-worded items with 5 response categories. The score ranges from 14 to 70. A higher score of WEMWBS represents higher mental well-being. The Cronbach’s alpha was 0.86 for WEMWBS.

#### Other variables

Mental status before delivery and support from family or social members were included as potential confounders. Previous mental status may confound because it is related to the perception of care during childbirth; it also becomes a risk factor for postpartum depression [10, 33]. Support from family or social members may confound because practical and emotional support from birth companions has been shown to positively affect the childbirth experience [34]. A low level of social support was one of the risk factors for postpartum depression [10]. We measured “support from family” and “support from social members” based on women’s perception toward the support, and the responses were Yes, No, or I don’t know. Socio-demographic factors and perinatal characteristics were also measured. These variables were used based on the questionnaire of the Nepal Demographic Health Survey in 2016 [14].

### Questionnaire

Questions for the PCMC scale, WEMWBS, and other variables were translated from English into Nepali by two Nepali researchers and then back-translated into English by two different Nepali researchers. All researchers had a background in health sciences. We conducted a pilot study to test all scales and questions among 20 women living in the Kathmandu district. These data were not included in the main study. After the pilot study, we revised several phrases. For example, for greater specificity, we modified the possible responses regarding baby’s birth weight from “1. being very large to 5. being very small” to “1. being very large (>4,500 g) to 5. being very small (<1,500 g)”. Additional file 1 shows the English version of the questionnaire used in this study.

### Data collection

We collected data from July to August 2019 using a structured questionnaire. We visited households of eligible women and conducted face-to-face interviews. We hired eight research assistants with public health backgrounds for the data collection. They received a one-day training session for data collection, research, and ethical considerations. Data were collected using a paper-based questionnaire and then entered into EpiData 3.1. The first author double-checked the entered data. Participants with missing data for PCMC, EPDS, or WEMWBS were excluded from the analyses. Missing data on socio-demographic factors and perinatal characteristics were replaced with “I don’t know” or “N/A.”

### Data analyses

We measured the differences in women’s characteristics between urban and rural areas using the Mann-Whitney test for age, chi-squared tests for other socio-demographic factors, and women’s perinatal characteristics. We performed the principal component analysis to estimate the household’s wealth score based on household wealth variables.

We used the score to categorize women into five wealth quintiles from the 1st (poorest) to the 5th (richest).

We calculated the means and standard deviations (SD) of the PCMC scale, EPDS, and WEMWBS. We compared the scores of each scale between urban and rural areas by using a t-test to observe whether there were any differences.

We performed a multiple regression analysis to analyze the association of the PCMC experience with the levels of maternal depressive symptoms and mental well-being, adjusting for other variables. The following variables were controlled for in the analysis: mother’s age, religion, caste/ethnicity, education, occupation, wealth quintile, baby’s age, the number of antenatal care (ANC) visits, baby’s sex, baby’s birth weight, parity, length of stay at the healthcare facility after delivery, husband/partner living elsewhere, delivery (vaginal delivery or cesarean section), complication during pregnancy or delivery, diagnosis of previous mental problems, anxiety/stress/depressive symptoms during pregnancy, unwanted pregnancy, and family support after delivery. We have excluded “friends/relatives/community support during pregnancy” and “friends/relatives/community support after delivery” because the variance inflation factors were 10 or higher to avoid possible multi-collinearity. We conducted bootstrapping for the multiple regression analysis considering a cluster effect in each ward. Moreover, we performed a simple regression analysis to show the unadjusted results. The significance level was set at 5%. All data analyses were performed using Stata/SE 15.

### Ethical considerations

In this study, we obtained ethical approval from the University of Tokyo Research Ethics Committee (Serial number: 2019088NI) and the Nepal Health Research Council (Reference number: 51). We obtained written informed consent before the interview. All the women voluntarily participated in this study, and we assured their confidentiality.

## Results

### Socio-demographic characteristics of women

In this study, we recruited 314 women from urban areas and 308 women from rural areas. Due to incomplete or missing data, we excluded 18 women from urban areas and 9 women from rural areas. In total, we included 595 (95.7%) women for data analyses.

Table 1 shows the socio-demographic characteristics of the women included in this study. The mean age was 24.8 years (SD 4.5) in urban areas and 23.2 years (SD 4.2) in rural areas. While 25.3% of women completed secondary education or higher in urban areas, only 16.7% completed it in rural areas. Regarding the wealth quintile, women who were categorized in the 4th (richer) or 5th (richest) quintile were 44.6% in urban areas and 35.5% in rural areas.

**Table 1:** Socio-demographic characteristics of women

Variables	Urban areas (n = 296)		Rural areas (n = 299)	
	**N**	**%**	**N**	**%**
**Age**	Mean = 24.8		Mean = 23.2	
≤ 19	36	12.2	53	17.7
20–24	112	37.8	148	49.5
25–29	101	34.1	71	23.8
30–34	37	12.5	22	7.4
≥ 35	10	3.4	5	1.7
**Religion**				
Hindi	245	82.8	261	87.3
Buddhism	36	12.2	27	9.0
Muslim	7	2.4	2	0.7
Christian	4	1.3	7	2.3
Other	4	1.3	2	0.7
**Caste / Ethnicity**				
Bharamin / Chhetri	88	29.7	106	35.5
Janajati	163	55.1	138	46.2
Dalit	31	10.5	48	16.1
Other	14	4.7	7	2.3
**Education**				
No formal education	34	11.5	33	11.0
Primary	54	18.2	74	24.8
Secondary	133	44.9	142	47.5
Higher	75	25.3	50	16.7
**Occupation**				
Housewife	214	72.3	219	73.2
Farmer	45	15.2	46	15.4
Business	29	9.8	20	6.7
Sell things	4	1.4	8	2.7
Others	4	1.4	6	2.1
**Wealth quintile**				
1. Poorest	45	15.2	74	24.8
2. Poorer	56	18.9	63	21.1
3. Middle	63	21.3	56	18.7
4. Richer	68	23.0	52	17.4
5. Richest	64	21.6	54	18.1

N = total number of women

### Perinatal characteristics of women

Table 2 shows women’s perinatal characteristics. More than 80% of women received ANC four times or more (83.8% in urban areas and 87.0% in rural areas) and had a vaginal delivery (94.3% in urban areas and 99.3% in rural areas). In urban areas, 79.4% of women lived with their husbands, while this was 88.6% in rural areas. Less than 5% of women experienced complications during the last delivery in both areas (4.7% in urban areas and 3.7% in rural areas).

**Table 2:** **Perinatal characteristics of women**

Variables	Urban areas (n = 296)		Rural areas (n = 299)		p-value
	**N**	**%**	**N**	**%**	
**Baby’s age**					**0.012**
1–3 months	69	23.3	45	15.1	
4–6 months	73	24.7	69	23.1	
7–9 months	64	21.6	60	20.1	
10–12 months	90	30.4	125	41.8	
**Baby’s sex**			(N/A = 1)		0.346
Boy	155	52.4	169	56.5	
Girl	141	47.6	129	43.1	
**ANC visits**					0.273
1–3 times	48	16.2	39	13.0	
≥ 4 times	248	83.8	260	87.0	
**Baby’s birth weight**	(N/A = 1)		(N/A = 1)		
4,000–4,500 g	6	2.0	8	2.7	0.957
2,500–3,999 g	279	94.3	279	93.3	
1,500–2,499 g	10	3.4	11	3.7	
**Length of stay at healthcare facility after delivery**					0.257
< 24 h	69	23.3	73	24.4	
24–48 h	182	61.5	169	56.5	
48–72 h	28	9.5	43	14.4	
≥ 72 h	17	5.7	14	4.7	
**Husband / partner**	(N/A = 1)		(N/A = 2)		**0.005**
Living together	235	79.4	265	88.6	
Staying elsewhere	60	20.3	32	10.7	
**Parity**					0.223
Primiparous	120	40.5	136	45.5	
Multiparous	176	59.5	163	54.5	
**Delivery**					**< 0.001**
Vaginal delivery	279	94.3	297	99.3	
Cesarean section	17	5.7	2	0.7	
**Complication during pregnancy or delivery**					**0.003**
No	266	89.9	286	95.7	
Yes	14	4.7	11	3.7	
I don’t know	16	5.4	2	0.6	
**Diagnosis of previous mental problems**					0.254
No	281	94.9	291	97.3	
Yes	13	4.4	6	2.0	
I don’t know	2	0.7	2	0.7	
**Anxiety/stress/ depressive symptoms during pregnancy**					0.927
No	244	82.4	250	83.6	
Yes	49	16.6	46	15.4	
I don’t know	3	1.0	3	1.0	
**Unwanted pregnancy**					0.608
No	269	90.9	268	89.6	
Yes	27	9.1	31	10.4	
**Family support after delivery**					0.784
Yes	292	98.7	293	98.0	
No / I don’t know	4	1.3	6	2.0	
**Friends/relatives/ community support after delivery**					0.159
Yes	296	100.0	297	99.3	
No / I don’t know	0	0	2	0.7	

N/A = not applicable; p-value is for Chi-squared test

### Scores of each scale

The mean score of the PCMC scale was higher in rural areas (66.7, SD 12.1) than in urban areas (63.3, SD 13.5) (*p* = 0.002). In EPDS, the mean score was 5.25 (SD 5.42) in urban areas, and was 5.04 (SD 4.59) in rural areas. The proportion of women with scores over the EPDS cut-off point was 12.2% in urban areas and 8.4% in rural areas (*p* = 0.126). The mean score of WEMWBS was 56.1 (SD 7.52) in urban areas and 58.1 (SD 7.27) in rural areas (*p* = 0.008). Additional file 2 shows the histogram for the scores of each scale.

### Association between PCMC experience and depressive symptoms

Table 3 shows the factors associated with depressive symptoms among women living in Dhading. In urban areas, the experience of better PCMC was associated with lower depressive symptom scores (unstandardized coefficient [B]= − 0.09, 95% CI: − 0.13, − 0.04). It means for each additional score increased in PCMC, the score of depressive symptoms decreased by an average of − 0.09 points, holding the other exposure variables constant. Among other variables, the poorest wealth quintile was associated with lower depressive symptom scores (B= − 2.55, 95% CI: − 3.81, − 1.28), compared to the middle quintile. It means the score of depressive symptoms was − 2.55 points lower for women with the poorest wealth quintile than for the reference group (middle wealth quintile). The following factors were significantly associated with higher depressive symptom scores: working as a farmer (B= 3.77, 95% CI: 1.47, 6.08, compared with housewife), staying at the healthcare facility after delivery for 48–72 h (B= 4.99, 95% CI: 2.54, 7.43), and longer than 72 h (B= 2.76, 95% CI: 0.11, 5.40) compared to staying for 24–48 h. In addition, having no formal education (B= 2.03, 95% CI: 0.10, 3.96, compared with secondary education), having visited ANC less than 4 times (B= 2.00, 95% CI: 0.04, 3.97), and having been diagnosed with mental problems previously (B= 4.41, 95% CI: 2.20, 6.60) were associated with higher depressive symptom scores, which was unique in urban areas.

**Table 3:** Factors associated with depressive symptoms among women living in Dhading

Variables	Urban areas						Rural areas					
	**Adjusted** ^a^			**Unadjusted** ^b^			**Adjusted**			**Unadjusted**		
	**B**	**95% CI**	**p-value**	**B**	**95% CI**	**p-value**	**B**	**95% CI**	**p-value**	**B**	**95% CI**	**p-value**
Person-centered maternity care	-0.09	-0.13, -0.04	**< 0.001**	-0.14	-0.18, -0.10	**< 0.001**	**-0.10**	**-0.15, -0.04**	**< 0.001**	-0.09	-0.13, -0.05	**< 0.001**
Age	-0.26	-1.00, 0.47	0.483	0.09	-0.05, 0.23	0.214	-0.01	-1.08, 0.15	0.983	-0.04	-0.16, 0.08	0.541
Religion (vs. Hindi)												
Buddhism	0.07	-1.45, 1.58	0.933	0.32	-1.56, 2.20	0.740	0.16	-1.49, 1.81	0.852	1.00	-0.82, 2.83	0.280
Muslim / Christian	-0.37	-2.70, 1.97	0.757	4.48	1.68, 7.28	**0.002**	-1.03	-2.78, 0.73	0.251	1.84	-0.94, 4.62	0.193
Caste / Ethnicity (vs. Janajati)												
Bharamin / Chhetri	-0.62	-1.93, 0.69	0.352	-0.22	-1.62, 1.19	0.761	0.66	-0.53, 1.85	0.279	0.09	-1.08, 1.25	0.886
Dalit	0.81	-1.27, 2.89	0.445	1.02	-1.07, 3.11	0.335	0.68	-1.08, 2.44	0.446	1.21	-0.30, 2.73	0.115
Education (vs. secondary)												
No formal	2.03	0.10, 3.96	**0.039**	4.54	2.56, 6.53	**< 0.001**	1.00	-0.50, 2.50	0.191	1.86	0.13, 3.61	**0.035**
Primary	0.30	-1.39, 1.99	0.728	1.36	-0.31, 3.03	0.109	0.28	-1.27, 1.83	0.726	0.76	-0.53, 2.05	0.250
Higher	0.36	-1.23, 1.95	0.655	-0.11	-1.60, 1.38	0.883	-0.82	-2.71, 1.07	0.395	0.84	-0.64, 1.32	0.836
Occupation (vs. Housewife)												
Farmer	3.77	1.47, 6.08	**0.001**	3.42	1.72, 5.13	**0.000**	2.33	0.88, 3.78	**0.002**	2.60	1.16, 4.04	**0.000**
Business / Sell things	-0.68	-2.74, 1.38	0.519	-0.90	-2.75, 0.95	0.339	0.41	-1.48, 2.29	0.673	0.04	-1.60, 1.67	0.964
Wealth quintile (vs. Middle)												
Poorest	-2.55	-3.81, -1.28	**< 0.001**	-2.71	-4.79, -0.64	**0.010**	-0.18	-1.49, 1.13	0.788	-0.75	-2.34, 0.84	0.355
Poorer	-1.28	-3.21, 0.65	0.195	-1.50	-3.45, 0.45	0.130	0.21	-0.99, 1.41	0.736	0.04	-1.61, 1.70	0.959
Richer	-0.42	-1.81, 0.97	0.556	-1.20	-3.06, 0.66	0.204	-1.26	-3.34, 0.82	0.236	-1.90	-3.64, -0.17	**0.031**
Richest	-0.71	-2.39, 0.97	0.408	-1.45	-3.33, 0.44	0.132	-0.06	-2.09, 1.96	0.951	-1.10	-2.82, 0.61	0.207
Baby’s age	-0.00	-0.01, 0.00	0.677	-0.01	-0.01, 0.00	0.053	-0.00	-0.01, 0.00	0.329	-0.00	-0.01, 0.00	0.155
ANC visit (vs. ≥ 4 times)												
1–3 times	2.00	0.04, 3.97	**0.046**	3.55	1.92, 5.19	**< 0.001**	-0.92	-1.90, 0.07	0.069	1.37	-0.18, 2.91	0.083
Baby’s sex (vs. Boy)												
Girl	0.20	-1.03, 1.43	0.748	0.00	-1.24, 1.25	0.995	-0.03	-0.96, 0.90	0.947	0.23	-0.83, 1.29	0.665
Baby’s birth weight (vs. 2,500–3,999 g)												
4,000–4,500 g	2.74	-1.99, 7.48	0.256	2.78	-1.57, 7.13	0.210	-2.28	-5.29, 0.74	0.139	-1.20	-4.43, 2.04	0.468
1,500–2,499 g	-1.30	-2.99, 0.39	0.131	4,75	1.35, 8,14	**0.006**	-0.78	-4.67, 3.10	0.692	-0.62	-3.39, 2.16	0.662
Delivery (vs. Vaginal delivery)												
Cesarean section	-1.09	-3.84, 1.67	0.438	0.98	-1.69, 3.66	0.471	-5.68	-8.60, -2.76	**< 0.001**	-2.56	-8.98, 3.86	0.433
Parity (vs. Multiparous)												
Primiparous	-0.45	-2.11, 1.20	0.591	-0.78	-2.04, 0.49	0.227	0.24	-1.13, 1.62	0.729	0.19	-0.86, 1.24	0.722
Length of stay at healthcare facility after delivery (vs. 24–48 h)												
< 24 h	1.22	-0.48, 2.93	0.159	1.28	-0.14, 2.69	0.077	-0.29	-1.10, 0.52	0.478	0.19	-1.04, 1.41	0.764
48–72 h	4.99	2.54, 7.43	**< 0.001**	6.43	4.40, 8.46	**< 0.001**	1.75	0.35, 3.15	**0.014**	2.60	1.11, 4.09	**0.001**
≥ 72 h	2.76	0.11, 5.40	**0.041**	3.56	1.02, 6.10	**0.006**	3.83	1.87, 5.80	**< 0.001**	4.74	2.31, 7.17	**< 0.001**
Husband / partner living elsewhere (vs. Living together)												
Staying elsewhere	-0.43	-1.04, 2.60	0.401	-0.84	-2.38, 0.70	0.285	2.15	0.21, 4.09	**0.030**	1.67	-0.02, 3.35	0.052
Complication during pregnancy or delivery (vs. No)												
Yes	0.78	-1.47, 3.06	0.491	1.68	-1.24, 4.60	0.258	-0.49	-2.43, 1.46	0.624	-0.52	-3.30, 2.26	0.715
Diagnosis of previous mental problems (vs. No)												
Yes	4.41	2.20, 6.60	**< 0.001**	4.56	1.58, 7.55	**0.003**	-1.87	-4.16, 0.42	0.109	-0.04	-3.78, 3.69	0.981
Anxiety / stress / depressive symptoms during pregnancy (vs. No)												
Yes	1.72	-0.57, 4.01	0.141	2.26	0.61, 3.92	**0.007**	2.89	1.20, 4.59	**0.001**	2.80	1.38, 4.22	**< 0.001**
Unwanted pregnancy (vs. No)												
Yes	0.18	-1.49, 1.85	0.834	-1.05	-3.21, 1.10	0.337	-0.36	-1.73, 1.01	0.605	-1.34	-3.06, 0.87	0.123
Family support after delivery (vs. Yes)												
No / I don’t know	-4.85	-16.04, 6.34	0.396	1.01	-4.37, 6.39	0.712	-6.19	-14.21, 1.83	0.130	-4.30	-8.00 -0.59	**0.023**

B = unstandardized coefficient; CI = Confidence Interval. The base level was set as the most frequent value within the choice, except for the wealth quintile. The base level of the wealth quintile was set at a middle value.

^a^ Adjusted: results of multiple regression analysis.

^b^ Unadjusted: results of simple regression analysis.

Likewise, the experience of better PCMC was associated with lower depressive symptom scores in rural areas (B= − 0.10, 95% CI: − 0.15, − 0.04). The following factors were associated with higher depressive symptom scores: working as a farmer (B= 2.33, 95% CI: 0.88, 3.78, compared to housewife), staying at the healthcare facility after delivery for 48–72 h (B= 1.75, 95% CI: 0.35, 3.15) and longer than 72 h (B= 3.83, 95% CI: 1.87, 5.80) compared to staying for 24–48 h. In addition, having a husband living elsewhere (B= 2.15, 95% CI: 0.21, 4.09), feeling anxious during pregnancy (B= 2.89, 95% CI: 1.20, 4.59), and delivering a baby with cesarean section (B= − 5.68, 95% CI: − 8.60, − 2.76, compared with vaginal delivery) were associated with higher depressive symptom scores, which was unique in rural areas.

### Association between PCMC experience and mental well-being

Table 4 shows the factors associated with mental well-being among women living in Dhading. In urban areas, the experience of better PCMC was associated with higher mental well-being scores (B= 0.30, 95% CI: 0.25, 0.35). Increased age was associated with higher mental well-being scores (B= 1.07, 95% CI: 0.14, 2.00), which was unique in urban areas.

**Table 4:** Factors associated with mental well-being among women living in Dhading

Variables	Urban areas						Rural areas					
	**Adjusted** ^a^			**Unadjusted** ^b^			**Adjusted**			**Unadjusted**		
	**B**	**95% CI**	**p-value**	**B**	**95% CI**	**p-value**	**B**	**95% CI**	**p-value**	**B**	**95% CI**	**p-value**
Person-centered maternity care	0.30	0.25, 0.35	**< 0.001**	0.33	0.27, 0.38	**< 0.001**	0.15	0.03, 0.27	**0.017**	0.11	0.04, 0.18	**0.001**
Age	1.07	0.14, 2.00	**0.024**	0.17	-0.03, 0.36	0.088	0.02	-0.18, 0.21	0.872	0.04	-0.16, 0.24	0.682
Religion (vs. Hindi)												
Buddhism	1.43	-1.13, 4.00	0.273	1.51	-1.12, 4.14	0.260	0.03	-4.45, 4.51	0.990	-1.73	-4.61, 1.16	0.240
Muslim / Christian	2.13	-4.09, 8.35	0.501	-3.50	-7.42, 0.42	0.080	-0.25	-3.41, 2.91	0.877	-3.45	-7.84, 0.94	0.124
Caste / Ethnicity (vs. Janajati)												
Bharamin / Chhetri	0.41	-1.25, 2.06	0.628	1.07	-0.89, 3.02	0.284	1.59	-0.48, 3.65	0.132	1.31	-0.53, 3.14	0.163
Dalit	-1.15	-4.95, 0.64	0.209	-1.09	-3.99, 1.81	0.460	0.03	-2.59, 2.65	0.982	-0.90	-3.28, 1.49	0.461
Education (vs. secondary)												
No formal	0.41	-2.98, 3.80	0.814	-3.02	-5.79, -0.24	**0.033**	-0.46	-3.28, 2.36	0.750	-2.39	-5.15, 0.37	0.089
Primary	-0.07	-1.59, 1.46	0.931	-0.27	-2.59, 2.06	0.822	0.65	-1.21, 2.52	0.493	-0.32	-2.36, 1.72	0.758
Higher	2.15	− 0.39, 4.68	0.097	3.20	1.12, 5.28	**0.003**	-0.47	-3.41, 2.47	0.754	1.06	-1.29, 3.41	0.374
Occupation (vs. Housewife)												
Farmer	-0.05	-2.36, 2.26	0.966	2.61	0.22, 5.00	**0.033**	0.06	-2.92, 3.04	0.971	1.07	-1.24, 3.38	0.363
Business / Sell things	1.52	-0.81, 3.85	0.201	3.63	1.04, 6.23	**0.006**	3.36	-0.39, 7.05	0.075	3.06	0.43, 5.68	**0.022**
Wealth quintile (vs. Middle)												
Poorest	0.40	-1.96, 2.77	0.738	0.04	-2.86, 2.94	0.976	-3.39	-7.13, 0.34	0.075	-3.05	-5.56, -0.53	**0.018**
Poorer	1.50	-0.44, 3.45	0.130	1.03	-1.70, 3.75	0.460	-2.33	-5.40, 0.75	0.138	-2.56	-5.17, 0.05	0.054
Richer	-1.23	-3.31, 0.86	0.248	-0.47	-3.07, 2.13	0.723	-2.05	-5.61, 1.51	0.259	-1.03	-3.76, 1.71	0.460
Richest	-0.14	-2.68, 2.40	0.916	0.82	-1.54, 3.73	0.413	-3.24	-6.83, 0.35	0.077	-0.53	-3.24, 2.18	0.700
Baby’s age	0.01	-0.00, 0.01	0.113	0.01	0.01, 0.02	**< 0.001**	-0.00	-0.01, 0.00	0.302	-0.00	-0.01, 0.01	0.598
ANC visit (vs. ≥ 4 times)												
1–3 times	-1.12	-3.00, 0.72	0.232	-4.03	-6.33, -1.74	**0.001**	1.74	-0.68, 4.15	0.159	-0.38	-2.84, 2.08	0.762
Baby’s sex (vs. Boy)												
Girl	-0.24	-1.84, 1.36	0.768	-0.26	-1.99, 1.47	0.767	-1.78	-3.51, -0.06	**0.042**	-2.20	-3.86, -0.54	**0.010**
Baby’s birth weight (vs. 2,500–3,999 g)												
4,000–4,500 g	-2.34	-10.65, 5.98	0.582	-5.29	-11.30, 0.72	0.084	-0.35	-7.23, 6.54	0.920	0.05	-5.08, 5.18	0.985
1,500–2,499 g	-1.87	-5.45, 1.71	0.305	-7.36	-12.05, -2.67	0.002	-2.43	-9.83, 4.97	0.520	-2.75	-7.15, 1.65	0.220
Delivery (vs. Vaginal delivery)												
Cesarean section	-0.72	-4.76, 3.33	0.728	-2.74	-6.43, 0.96	0.146	7.20	-0.75, 15.15	0.076	5.46	-4.69, 15.61	0.290
Parity (vs. Multiparous)												
Primiparous	0.25	-1.63, 2.13	0.794	0.20	-1.56, 1.95	0.826	-1.11	-2.66, 0.45	0.164	-0.30	-1.96, 1.37	0.726
Length of stay at healthcare facility after delivery (vs. 24–48 h)												
< 24 h	1.04	-0.58, 2.65	0.207	0.18	-1.85, 2.20	0.862	0.90	-1.10, 2.89	0.377	0.75	-1.24, 2.73	0.461
48–72 h	-1.25	-3.48, 0.98	0.272	-5.67	-8.58, -2.77	**0.000**	-0.63	-3.15, 1.89	0.624	-0.95	-3.38, 1.47	0.440
≥ 72 h	-2.94	-7.24, 1.36	0.181	-5.57	-9.20, -1.94	**0.003**	-3.15	-8.68, 2.38	0.264	-4.83	-8.77, -0.88	**0.017**
Husband / partner living elsewhere (vs. Living together)												
Staying elsewhere	-0.31	-2.36, 1.74	0.766	0.35	-1.80, 2.49	0.752	-2.58	-4.60, -0.56	**0.012**	-1.13	-3.81, 1.54	0.406
Complication during pregnancy or delivery (vs. No)												
Yes	-1.66	-6.84, 3.52	0.530	-0.49	-4.55, 3.57	0.813	1.86	-2.16, 5.89	0.364	1.91	-2.49, 6.30	0.394
Diagnosis of previous mental problems (vs. No)												
Yes	-2.50	-7.09, 2.09	0.285	0.69	-3.51, 4.90	0.747	-2.85	-6.92, 1.21	0.169	-2.46	-8.36, 3.45	0.414
Anxiety / stress / depressive symptoms during pregnancy (vs. No)												
Yes	-0.61	-2.33, 1.11	0.488	-1.65	-3.96, 0.67	0.162	1.05	-2.15, 4.25	0.520	1.35	-0.94, 3.64	0.247
Unwanted pregnancy (vs. No)												
Yes	-1.27	-5.06, 2.52	0.511	0.37	-2.62, 3.36	0.808	-3.65	-5.53, -1.77	**< 0.001**	-2.75	-5.44, -0.04	**0.046**
Family support after delivery (vs. Yes)												
No / I don’t know	-2.96	-10.53, 4.60	0.443	-2.90	-10.36, 4.56	0.445	7.30	-1.87, 16.47	0.119	5.03	-0.85, 10.91	0.094

B = unstandardized coefficient; CI = Confidence Interval. The base level was set as the most frequent value within the choice, except for the wealth quintile. The base level of the wealth quintile was set at a middle value.

^a^ Adjusted: results of multiple regression analysis.

^b^ Unadjusted: results of simple regression analysis.

In rural areas, the experience of better PCMC was associated with higher mental well-being scores (B= 0.15, 95% CI: 0.03, 0.27). However, the following factors were also associated with lower mental well-being scores: delivering a baby girl (B= − 1.78, 95% CI: − 3.51, − 0.06), having a husband living elsewhere (B= − 2.58, 95% CI: − 4.60, − 0.56), and unwanted pregnancy (B= − 3.65, 95% CI: − 5.53, − 1.77).

## Discussion

In this study, women who received better PCMC had lower depressive symptom scores and were more likely to have higher mental well-being after delivery, regardless of urban or rural settings. Through the analysis, other factors were also found to be associated with mental health status. Although we expected to see differences in the association of PCMC with mental health outcomes between the urban and rural areas, we found similar results in both areas.

In this study, women who experienced better PCMC had lower depressive symptom scores, both in urban and rural areas. Women’s satisfaction with the medical staff members’ attitudes during childbirth was negatively associated with postpartum post-traumatic stress in Turkey [35]. In Kenya, PCMC improved women’s self-efficacy and positively affected maternal and child health [36]. PCMC during delivery decreases anxiety or negative feeling toward childbirth. However, disrespect and abusive care experiences during childbirth were associated with women’s postpartum depression in Brazil [37]. These findings are consistent with the results of the current study. Though the association between PCMC and depressive symptoms was statistically significant, the effect size was relatively small. One reason could be that depressive symptoms are complex, and various factors influence them. Another reason might be that we assessed PCMC through self-reported scores, which could underestimate the inappropriateness of care. Nevertheless, when creating a favorable delivery environment, it is required that healthcare providers can provide adequate PCMC.

This study also found that women who experienced better PCMC were more likely to have higher mental well-being after delivery in urban and rural settings. Building a favorable relationship between mothers and midwives during childbirth was essential for mothers to have a positive birth experience [38]. In Iran, positive childbirth experiences improved women’s self-efficacy and self-esteem after delivery [39]. Furthermore, in Rwanda, sufficient support and adequate maternity care increased the health-related quality of life for women who delivered within 1–13 months [40]. Women who trust medical staff members might seek healthcare when they encounter health problems after delivery and thus receive support. Therefore, PCMC during delivery contributes to improving women’s well-being after delivery by providing positive childbirth experiences and promoting sufficient support after delivery.

Initially, we expected that the associations of PCMC with depressive symptoms and mental well-being in urban areas would be different from those in rural areas. Previous studies in Nepal reported that 17.1–30.3% of mothers had depressive symptoms in urban areas, while 9.8% of women were distressed in rural areas [19, 20, 41, 42]. The mean PCMC scores also varied depending on the settings. For example, one study reported that urban Kenya presented the highest mean score, while rural Ghana showed the lowest mean score [28]. Additionally, a previous systematic review showed that the mismatch between birth expectation and experience can decrease birth satisfaction and possibly increase postnatal post-traumatic stress disorder [43]. Since urban areas have more facility type options, such as private hospitals vs. public facilities, this might increase expectations and consequently, the association between mothers’ delivery experience and maternal mental health would be stronger in urban areas than in rural areas. Since Nepal has a multiethnic and multicultural society [14], we hypothesized that the setting might influence the association between PCMC and mental health. However, we attained similar results for both urban and rural areas, that is, although the magnitudes were not the same, there was a negative association between PCMC and depressive symptoms and a positive association between PCMC and mental well-being in both urban and rural areas. This may be because we compared urban and rural areas within the same district. We classified areas called “municipalities” as urban areas and areas classified “rural municipalities” as rural areas based on the Nepalese administrative categorization. Therefore, those areas included in this study may not necessarily represent urban or rural settings. Another reason could be that urban-rural settings may not matter regarding PCMC, as it is based on women’s customs and culture [44]. Therefore, PCMC can improve maternal mental health regardless of the settings. Further research is required to determine how the living settings influence PCMC and maternal mental health.

Along with PCMC, factors such as being a farmer, a longer stay in a healthcare facility after delivery, and previous mental problems were associated with depressive symptoms in both urban and rural areas. Working women were more likely to have postnatal depression in India [45]. Women working as farmers might have difficulties when working and parenting concurrently. Clinical problems may extend the length of stay [46, 47]. Adverse reproductive health was associated with depressive symptoms [48]. Furthermore, socio-psychological problems were risk factors for developing depressive symptoms after delivery [47, 49].

In urban areas, lower education levels, fewer ANC visits, and the poorest wealth quintile were factors associated with depressive symptoms, while increased age was the unique factor associated with higher mental well-being. Women’s education level was one of the barriers to using adequate ANC in India [50]. Fewer ANC visits increased the risk of depressive symptoms due to a lower chance of consulting and receiving support [20]. However, women in the poorest wealth quintile had lower depressive symptom scores in this study. This could be because the wealth quintile did not include household income and overseas remittances or because the women got special support. In addition, increased age was one of the determinants of decision-making toward sexual and reproductive health in Nepal [51].

In rural areas, delivering a baby girl, having a husband living elsewhere, an unwanted pregnancy, and cesarean section were factors associated with mental well-being. Women reported some pressure to have sons, which was related to virilocal or patrilineal inheritance in Nepal [52]. In Vietnam, negative family responses to the baby reduced mental well-being [53]. Poor relationships with husbands or a lack of partner support were also risk factors for depressive symptoms [54]. Husband’s involvement was important for maternal health and safe childbirth [55]. Experience of unwanted pregnancy was also associated with maternal complications in rural India [56] and reported as a risk factor for postpartum depressive symptoms [57]. Cesarean section was shown to increase the risk of depressive symptoms in Nepal in a previous study [20]. However, we could not verify the association between depressive symptoms and cesarean section from our results since only a few women delivered through a cesarean section in rural areas.

This study had several limitations: First, participating women were not randomly recruited from all the eligible women in the selected areas. Due to our sampling method, we might have missed certain eligible women who were not introduced to us by local people. Second, this is a cross-sectional study, and the possibility of reverse causality exists for women’s delivery care experience and maternal mental health. Many factors are associated with childbirth care experiences and mental health, and unknown confounders should be considered. Furthermore, recall of childbirth experiences or mental status can change over time. Third, we conducted a one-day training for the research but did not measure inter-rater reliability. Despite some limitations, this is one of the few studies on the association between PCMC experiences and maternal mental health after delivery in Nepal. Healthcare facilities require further improvement regarding PCMC aspects, especially during childbirth care. For example, healthcare staff is required to have more effective communication, pay attention to women’s needs and feelings, and maintain safe facilities. Such improvements in PCMC may contribute to better maternal mental health. The findings may have implications for constructing better healthcare systems in Nepal and in other resource-limited settings.

## Conclusions

This study showed that PCMC during childbirth benefits women’s maternal mental health after delivery. Healthcare facilities are required to improve quality care by increasing the awareness of PCMC aspects. Providing suitable and equitable maternity care is important and can be done by considering the various backgrounds of care users. Further research is required to determine better PCMC for women to build better quality care.

## Electronic supplementary material

Below is the link to the electronic supplementary material.

**Additional File 1:** Questionnaire

**Additional File 2:** Histogram for each scale

**Additional File 3:** Datasets

## Data Availability

The datasets supporting the conclusions of this article are included within the article (and its additional files; see Additional file 3).

## Abbreviations

ANC: Antenatal care
B: Unstandardized coefficient
CI: Confidence Interval
EPDS: The Edinburgh Postnatal Depression Scale
N/A: Not applicable
PCMC: Person-centered maternity care
SD: Standard deviations
WEMWBS: The Warwick-Edinburgh Mental Wellbeing Scale
