# Supplementary material for: Association between the person-centered maternity care experience and mental health after delivery in urban and rural Dhading, Nepal: a cross-sectional study
Source: BMC Pregnancy Childbirth. 2023 May 30;23:398. doi: 10.1186/s12884-023-05709-z (PMC10228024; doi:10.1186/s12884-023-05709-z)
Supplement: Supplementary file 1 — Additional File 1: Questionnaire [file 12884_2023_5709_MOESM1_ESM.docx]

**the Person-Centered Maternity Care scale (PCMC scale)**

| **Now I am going to ask you some questions about your experiences in the health facility during your last delivery. Remember that all the questions in this section refer specifically to the time you were in the health facility for this last delivery. Also, know that everything you**  **tell me is confidential and will not be shared with the health facility.** | | | | | | | | | | | | | |
| --- | --- | --- | --- | --- | --- | --- | --- | --- | --- | --- | --- | --- | --- |
| #1: How did you feel about the amount of time you waited? Would you say it was very short, somewhat short, somewhat long, or very long? | Very short | | Somewhat short | | | | | Somewhat long | | | | Very long | |
|  | **0** | | **1** | | | | | **2** | | | | **3** | |
| #2: During your time in the health facility did the doctors, nurses, or other health care providers introduce themselves to you when they first came to see you? If yes: Was it a few of them, most of them, or all of them? | No, none of them | | Yes, a few  of them | | | | | Yes, most  of them | | | | Yes, all  of them | |
|  | **0** | | **1** | | | | | **2** | | | | **3** | |
| **Now I will ask you some questions about how you were treated at the health facility. Tell me if the following things happened all the time, most of the time, a few times, or it never happened. You can say a few times if it happened one or two times, and most of the time will be if it happened 3 or more times, but not always. For some questions I will ask specifically if something occurred during labor, delivery, or after delivery. If I do not specify, please answer based on your experiences during the entire time you were in the facility from labor till discharge.** | | | | | | | | | | | | | |
| #3: Did the doctors, nurses, or other health care providers call you by your name? | No,  never | | Yes, a  few times | | | | | Yes, most  of the time | | | | Yes, all  the time | |
|  | **0** | | **1** | | | | | **2** | | | | **3** | |
| #4: Did the doctors, nurses, or other staff at the facility treat you with respect? | **0** | | **1** | | | | | **2** | | | | **3** | |
| #5: Did the doctors, nurses, and other staff at the facility treat you in a friendly manner? | **0** | | **1** | | | | | **2** | | | | **3** | |
| #6: During examinations in the labor room, were you covered up with a cloth or blanket or screened with a curtain so that you did not feel exposed? | **0** | | **1** | | | | | **2** | | | | **3** | |
| #7: Do you feel like your health information was or will be kept confidential at this facility? | **0** | | **1** | | | | | **2** | | | | **3** | |
| #8: Did you feel like the doctors, nurses or other staff at the facility involved you in decisions about your care? | No,  never | Yes, a  few times | | | | Yes, most  of the time | | | | Yes, all  the time | | | Did not  have to  make any  decisions |
|  | **0** | **1** | | | | **2** | | | | **3** | | | **4** |
| #9: Did the doctors, nurses or other staff at the facility ask your permission/consent before doing procedures on you? | No,  never | | Yes, a  few times | | | | | Yes, most  of the time | | | | Yes, all  the time | |
|  | **0** | | **1** | | | | | **2** | | | | **3** | |
| #10: During the delivery, do you feel like you were able to be in the position of your choice? | No,  never | | Yes, for a  short time | | | | | Yes, most  of the time | | | | Yes, all  the time | |
|  | **0** | | **1** | | | | | **2** | | | | **3** | |
| #11: Did the doctors, nurses or other staff at the facility speak to you in a language you could understand? | No,  never | | Yes, a  few times | | | | | Yes, most  of the time | | | | Yes, all  the time | |
|  | **0** | | **1** | | | | | **2** | | | | **3** | |
| #12: Did the doctors and nurses explain to you why they were doing examinations or procedures on you? | **0** | | **1** | | | | | **2** | | | | **3** | |
| #13: Did the doctors and nurses explain to you why they were giving you any medicine? | No,  never | Yes, a  few times | | | | Yes, most  of the time | | | | Yes, all  the time | | | Did not  get any  medicine |
|  | **0** | **1** | | | | **2** | | | | **3** | | | **4** |
| #14: Did the doctors and nurses at the facility talk to you about how you were feeling? | No,  never | | | Yes, a  few times | | | Yes, most  of the time | | | | Yes, all  the time | | |
|  | **0** | | | **1** | | | **2** | | | | **3** | | |
| #15: Did the doctors, nurses or other staff at the facility try to understand your anxieties and fears? | No,  never | Yes, a  few times | | | Yes, most  of the time | | | | Yes, all  the time | | | | Did not  have any  anxieties  or fears |
|  | **0** | **1** | | | **2** | | | | **3** | | | | **4** |
| #16: Did you feel you could ask the doctors, nurses or other staff at the facility any questions you had? | Very short | | | Somewhat short | | | Somewhat long | | | | Very long | | |
|  | **0** | | | **1** | | | **2** | | | | **3** | | |
| #17: Were you allowed to have someone you wanted (from outside of staff at the facility, such as family or friends) to stay with you during labor? | No,  never | Yes, a  few times | | | Yes, most  of the time | | | | Yes, all  the time | | | | I did not  want  someone to  stay with me |
|  | **0** | **1** | | | **2** | | | | **3** | | | | **4** |
| #18: Were you allowed to have someone you wanted to stay with you during delivery? | **0** | **1** | | | **2** | | | | **3** | | | | **4** |
| #19: When you needed help, did you feel the doctors, nurses or other staff at the facility paid attention? | No,  never | | | Yes, a  few times | | | Yes, most  of the time | | | | Yes, all  the time | | |
|  | **0** | | | **1** | | | **2** | | | | **3** | | |
| #20: Do you feel the doctors or nurses did everything they could to help control your pain? | **0** | | | **1** | | | **2** | | | | **3** | | |
| #21: Did you feel the doctors, nurses, or other health providers shouted at you, scolded, insulted, threatened, or talked to you rudely? (If yes) will you say this happened once, a few times, or many times? | No, never | | | Yes, once | | | Yes, a few times | | | | Yes, many times | | |
|  | **0** | | | **1** | | | **2** | | | | **3** | | |
| #22: Did you feel like you were treated roughly like pushed, beaten, slapped, pinched, physically restrained, or gagged? (If yes) will you say this happened once, a few times, or many times? | **0** | | | **1** | | | **2** | | | | **3** | | |
| #23: Do you think there was enough health staff in the facility to care for you? | No,  never | | | Yes, a  few times | | | Yes, most  of the time | | | | Yes, all  the time | | |
|  | **0** | | | **1** | | | **2** | | | | **3** | | |
| #24: Did you feel the doctors, nurses or other staff at the facility took the best care of you? | **0** | | | **1** | | | **2** | | | | **3** | | |
| #25: Did you feel you could completely trust the doctors, nurses or other staff at the facility with regards to your care? | **0** | | | **1** | | | **2** | | | | **3** | | |
| **The next set of questions are about the health facility environment** | | | | | | | | | | | | | |
| #26: Thinking about the labor and postnatal wards, did you feel the health facility was crowded? | No, never | | | Yes, once | | | Yes, a few times | | | | Yes, many times | | |
|  | **0** | | | **1** | | | **2** | | | | **3** | | |
| #27: Thinking about the wards, washrooms and the general environment of the health facility, will you say the facility was very clean, clean, dirty, or very dirty? | Very dirty | | | Dirty | | | Clean | | | | Very clean | | |
|  | **0** | | | **1** | | | **2** | | | | **3** | | |
| #28: Was there water in the facility? | No,  never | | | Yes, a  few times | | | Yes, most  of the time | | | | Yes, all  the time | | |
|  | **0** | | | **1** | | | **2** | | | | **3** | | |
| #29: Was there electricity in the facility? | **0** | | | **1** | | | **2** | | | | **3** | | |
| #30: In general, did you feel safe in the health facility? | **0** | | | **1** | | | **2** | | | | **3** | | |

**Edinburgh Postnatal Depression Scale (EPDS)**

As you have recently had a baby, we would like to know how you are feeling. Please check the answer that comes closest to how you have felt **IN THE PAST 7 DAYS**, not just how you feel today.

Here is an example, already completed.

I have felt happy:

□ Yes, all the time This would mean: “I have felt happy most of the time” during the

☑ Yes, most of the time past week. Please complete the other questions in the same way.

□ No, not every often

□ No, not at all

**In the past 7 days:**

1. **I have been able to laugh and see the funny side of things.**

□ As much as I always could

□ Not quite so much now

□ Definitely not so much now

□ Not at all

1. **I have looked forward with enjoyment to things**

□ As much as I ever did

□ Rather less than I used to

□ Definitely less than I used to

□ Hardly at all

1. **I have blamed myself unnecessarily when things went wrong**

□ Yes, most of the time

□ Yes, some of the time

□ Not very often

□ No, never

1. **I have been anxious or worried for no good reason**

□ No, not at all

□ Hardly ever

□ Yes, sometimes

□ Yes, very often

**5, I have felt scared or panicky for no very good reason**

□ Yes, quite a lot

□ Yes, sometimes

□ No, not much

□ No, not at all

**6, Things have been getting on top of me**

□ Yes, most of the time I haven’t been able to cope at all

□ Yes, sometimes I haven’t been coping as well as usual

□ No, most of the time I have coped quite well

□ No, I have been coping as well as ever

**7, I have been so unhappy that I have had difficulty sleeping**

□ Yes, most of the time

□ Yes, sometimes

□ Not very often

□ No, not at all

**8, I have felt sad or miserable**

□ Yes, most of the time

□ Yes, quite often

□ Not very often

□ No, not at all

**9, I have been so unhappy that I have been crying**

□ Yes, most of the time

□ Yes, quite often

□ Only occasionally

□ No, never

**10, The thought of harming myself has occurred to me**

□ Yes, quite often

□ Sometimes

□ Hardly ever

□ Never

**The Warwick-Edinburgh Mental Well-being Scale (WEMWBS)**

Below are some statements about feelings and thoughts.

Please tick the box that best describes your experience of each over the last 2 weeks.

| **Statements** | **None of the time** | **Rarely** | **Some of the time** | **Often** | **All of the time** |
| --- | --- | --- | --- | --- | --- |
| 1. I’ve been feeling optimistic about the future | 1 | 2 | 3 | 4 | 5 |
| 1. I’ve been feeling useful | 1 | 2 | 3 | 4 | 5 |
| 1. I’ve been feeling relaxed | 1 | 2 | 3 | 4 | 5 |
| 1. I’ve been feeling interested in other people | 1 | 2 | 3 | 4 | 5 |
| 1. I’ve had energy to spare | 1 | 2 | 3 | 4 | 5 |
| 1. I’ve been dealing with problems well | 1 | 2 | 3 | 4 | 5 |
| 1. I’ve been thinking clearly | 1 | 2 | 3 | 4 | 5 |
| 1. I’ve been feeling good about myself | 1 | 2 | 3 | 4 | 5 |
| 1. I’ve been feeling close to other people | 1 | 2 | 3 | 4 | 5 |
| 1. I have been feeling   Confident | 1 | 2 | 3 | 4 | 5 |
| 1. I’ve been able to make up my own mind about things | 1 | 2 | 3 | 4 | 5 |
| 1. I’ve been feeling loved | 1 | 2 | 3 | 4 | 5 |
| 1. I’ve been interested in new things | 1 | 2 | 3 | 4 | 5 |
| 1. I’ve been feeling cheerful | 1 | 2 | 3 | 4 | 5 |

**Questions about your background**

This questionnaire asks your background. All the information that you provide will be treated as strictly confidential and will only be seen by the research team. Thank you very much for your help.

**・Socio-demographic factors**

| How old are you? |  |
| --- | --- |
| What is your religion? | - Hindi - Buddhist - Muslim - Kiart - Christian - Other ( ) |
| What is your caste/ethnicity? |  |
| Have you ever attended school? | □ Yes  □ No |
| What is the highest grade you have completed? |  |
| What is your occupation? That is, what kind of work do you mainly do? | □ Housewife  □ Farmer  □ Business  □ Sell things  □ Other ( ) |
| What kind of materials are used for your house wall? | □ No walls  □ Cane / palm / trunks  □ Mud / sand  □ Bamboo with mud  □ Stone with mud  □ Plywood  □ Cardboard  □ Reused wood  □ Metal / Galvanized sheet  □ Cement  □ Stone with lime / cement  □ Bricks  □ Cement blocks  □ Wood planks / shingles  □ Other ( ) |
| What is the main material of the floor of the dwelling? | □ Earth / Sand  □ Dung  □ Wood planks  □ Palm / bamboo  □ Parquet of polished wood  □ Vinyl of asphalt strips  □ Ceramic tiles  □ Cement  □ Carpet  □ Other ( ) |
| What is the main material of the roof of the dwelling? | □ No roof  □ Thatch / palm leaf  □ Rustic mat  □ Palm / bamboo  □ Wood planks  □ Cardboard  □ Metal / Galvanized sheet  □ Wood  □ Calamine / cement fiber  □ Ceramic tile  □ Cement  □ Roofing shingles  □ Other ( ) |
| What is the main source of drinking water for members of your household? | □ Piped into dwelling  □ Piped to yard / plot  □ Piped to neighbor  □ Public tap / standpipe  □ Tube well of borehole  □ Protected well  □ Unprotected well  □ Protected spring  □ Unprotected spring  □ Rainwater  □ Tanker truck  □ Cart with small tank  □ Surface water  (river/dam/lake/pond/stream/canal)  □ bottle water  □ Other ( ) |
| What kind of toilet facility do members of your household usually use? | □ Flush to piped sewer system  □ Flush to septic tank  □ Flush to pit latrine  □ Flush to somewhere else  □ Flush, Don’t know where  □ Ventilated improved pit latrine  □ Pit latrine with slab  □ Pit latrine without slab / open pin  □ Composting toilet  □ Bucket toilet  □ Hanging toilet/hanging latrine  □ No facility/ bush /field  □ Other ( ) |
| Do you share this toilet facility with other households? | □ Yes  □ No |
| What type of fuel does your household mainly use for cooking? | □ Electricity  □ LPG  □ Nature gas  □ Biogas  □ Kerosene  □ Coal, lignite  □ Wood  □ Straw / shrubs / grass  □ Agricultural crop  □ Animal dung  □ No food cooked in household  □ Other ( ) |
| Does any member of this household have a bank account / cooperative or other saving accounts? | □ Yes  □ No |
| How many rooms in this household are used for sleeping? | ( )rooms |
| How many of the following animals do your household own?  (multiple choice) | - Milk cows or bulls? - Buffalo? - Horses, donkeys, or mules? - Goas? - Sheep? - Chickens or other poultry? - Ducks? - Pigs? - Yaks? |
| Does any member of your household own any agricultural land? | □ Yes  □ No |
| Does your household have:  (multiple choice) | □ Electricity?  □ A radio?  □ A television?  □ A non-mobile telephone?  □ A computer?  □ A refrigerator?  □ A table?  □ A chair?  □ A bed?  □ A sofa?  □ A cupboard?  □ A clock?  □ A fan?  □ A invertor?  □ A dhiki / janto? |
| Does any member of this household own:  (multiple choice) | □ A watch?  □ A mobile phone?  □ A bicycle / rickshaw?  □ A motorcycle or motor scooter?  □ An animal-drawn cart?  □ A car / truck / tractor?  □ A three-wheel tempo? |

**・Perinatal characteristics**

| Which type of facility did you deliver? | □ Higher level hospital  □ District hospital  □ Primary health care center  □ Health post |
| --- | --- |
| Is your baby a boy or a girl? | □ Boy  □ Girl |
| Did you see anyone for antenatal care for this pregnancy? | □ Yes  □ No |
| How many times did you receive antenatal care during this pregnancy? | □ None  □ Yes (　　　　　　　　　　　)  □ I don’t know |
| When your baby was born, was he/she: | □ Very large (> 4,500 g)  □ Larger than average (4,000 g – 4,500 g)  □ Average (2,500 g – 3,999 g)  □ Smaller than average (1,500 g – 2,499 g)  □ Very small (< 1,500 g) |
| After your baby was delivered, how long did you stay there? | □ Hours ( )  □ Days ( )  □ Weeks ( )  □ Don’t know |
| Is your husband/partner living with you now or is he staying elsewhere? | □ Living with him  □ Staying elsewhere |
| How many children have you delivered? | □ primiparity  □ more than two ( times) |
| Did you deliver the child by Caesarean section or vaginal delivery? | □ Caesarean section  □ Vaginal delivery |
| Have you ever experienced a complication during pregnancy or delivery? | - Yes ( ) - No |
| When you got pregnant, did you want to get pregnant at that time? | □ Yes  □ No  □ I don’t know |

**・Previous mental status and support**

| Did you experienced or diagnosed as mental problems before? | □ Yes  □ No |
| --- | --- |
| Did you often feel anxiety, depression, sadness, or scary during pregnancy? | □ Yes  □ No  □ I don’t know |
| Did your family support well during your pregnancy? | □ Yes  □ No  □ I don’t know |
| Did your family support well during your delivery in the facility? | □ Yes  □ No  □ I don’t know |
| Did your family support well after delivery? | □ Yes  □ No  □ I don’t know |
| Did your friends, relatives, or community members support you during your pregnancy? | □ Yes  □ No  □ I don’t know |
| Do your friends, relatives, or community members support you after delivery? | □ Yes  □ No  □ I don’t know |

Date ………………………………

ID ………………………………
