# Supplementary material for: Association between the person-centered maternity care experience and mental health after delivery in urban and rural Dhading, Nepal: a cross-sectional study
Source: BMC Pregnancy Childbirth. 2023 May 30;23:398. doi: 10.1186/s12884-023-05709-z (PMC10228024; doi:10.1186/s12884-023-05709-z)
Supplement: Supplementary file 2 — Additional File 2: Histogram for each scale [file 12884_2023_5709_MOESM2_ESM.docx]

**Additional file 2: The histogram for each scale**

**PCMC scale**

**EPDS**

**WEMWBS**
